# Socioeconomic costs incurred by parents of children with inflammatory bowel diseases

**DOI:** 10.1007/s00431-025-06639-y

**Published:** 2025-11-23

**Authors:** Piotr Marczyński, Andrzej Wasilewski, Sylwiusz Kontek, Adrian Kasprzak, Katarzyna Akutko, Tomasz Pytrus

**Affiliations:** 1 Wroclaw Medical University, Wroclaw, Poland; 2 2nd Clinical Department of Paediatrics, Gastroenterology and Nutrition, Wroclaw Medical University, Wroclaw, Poland

**Keywords:** IBD, Costs, Paediatrics, Socioeconomic, Crohn, Ulcerative colitis

## Abstract

The aim of this study was to assess the socio-economic burden on families of children diagnosed with inflammatory bowel diseases (IBD), particularly ulcerative colitis (UC), and Crohn’s disease (CD). The study was conducted at the 2nd Clinic of Paediatrics, Gastroenterology and Nutrition of Wroclaw Medical University. Anonymous questionnaires were completed by parents of paediatric IBD patients. The survey collected demographic, socio-economic, and treatment-related data. The study identified both material and non-material costs associated with IBD care. These included frequent private medical visits, medication, diet modifications, and transportation. Average monthly travel expenses were PLN 215.9 for UC and PLN 302.3 for CD (PLN = Polish zloty, 1 PLN = 0.25 euro). Limited access to paediatric gastroenterologists, especially in remote areas, led many families to seek private care, with associated monthly costs averaging PLN 312.3 for UC and PLN 513.3 for CD. Additionally, parents reported frequent work absences due to caregiving responsibilities, further impacting income and quality of life.

*Conclusion*: Childhood IBD imposes a considerable financial and psychosocial burden on families. The findings underscore the need for improved access to public healthcare services and financial support mechanisms to reduce the strain on affected families.

**Table:** 

**What Is Known:** *• Families of children with IBD (ulcerative colitis, Crohn’s disease) face significant costs related to medical care and lifestyle changes.* *• Limited access to specialised paediatric gastroenterologists and frequent absences from work add to the overall family burden.* **What Is New:** *• This study quantifies the monthly out-of-pocket travel and private care costs for paediatric IBD in Poland (PLN 215.9–513.3), previously rarely documented.* *• The findings highlight significant non-material impacts, such as caregiver work absences, further reducing family quality of life.*

**What Is Known:**

*• Families of children with IBD (ulcerative colitis, Crohn’s disease) face significant costs related to medical care and lifestyle changes.*

*• Limited access to specialised paediatric gastroenterologists and frequent absences from work add to the overall family burden.*

**What Is New:**

*• This study quantifies the monthly out-of-pocket travel and private care costs for paediatric IBD in Poland (PLN 215.9–513.3), previously rarely documented.*

*• The findings highlight significant non-material impacts, such as caregiver work absences, further reducing family quality of life.*

## Introduction

Inflammatory bowel diseases (IBD) are conditions in which chronic inflammation of the gastrointestinal tract occurs with characteristic periods of remission and recurrence [1]. Symptoms that most often characterize the mentioned diseases are diarrhoea, also with blood, abdominal pain, anaemia, or weight loss [2]. The most common IBD are ulcerative colitis (UC) and Crohn’s disease (CD). The aetiology of IBD has not been fully elucidated. However, it is known to be complex and multifactorial. Among others, genetic, immunological, and environmental factors are involved in the development of these diseases [1]. An estimated 30% of diagnoses are made before the age of 20 [3]. The care of paediatric patients affected by IBD entails numerous costs and challenges. IBD, due to their characteristics, require regular monitoring and the use of medications to maintain remission. This results in parents incurring material costs and devoting their time to activities related to the treatment of their children, such as visits to clinical centres or private specialists. It has been proven that IBD, especially in the active stage, reduces quality of life, which is reflected in the psychological health of patients [4]. Paediatric patients are more prone to require hospitalization for IBD than adults, most often in the presence of a parent. This increases caregiver absenteeism and reduces family income. In order to maintain remission, in addition to the use of newer and more effective drugs, it is also recommended to maintain a proper diet, which helps reduce inflammation in the intestines. However, it also increases the expenses incurred in maintaining the child's well-being [5, 6]. One of the most efficient therapies used to treat these diseases is a biological treatment in which we use drugs such as infliximab or adalimumab. Such treatment entails many benefits but requires more frequent visits to clinical centres [6–8]. The purpose of this study was to discover the difficulties faced by parents of children with IBD and to determine the approximate costs associated with caring for the paediatric patient.

## Materials and methods

### Study design

The study was conducted via questionnaire on parents of children with IBD treated in the second Clinic of Paediatrics, Gastroenterology and Nutrition of Wroclaw Medical University, Poland, and lasted from January to October 2024. In accordance with the Helsinki Declaration, approval was obtained from the bioethics committee at the Wroclaw Medical University No. KB 508/2023N.

**Fig. 1:**
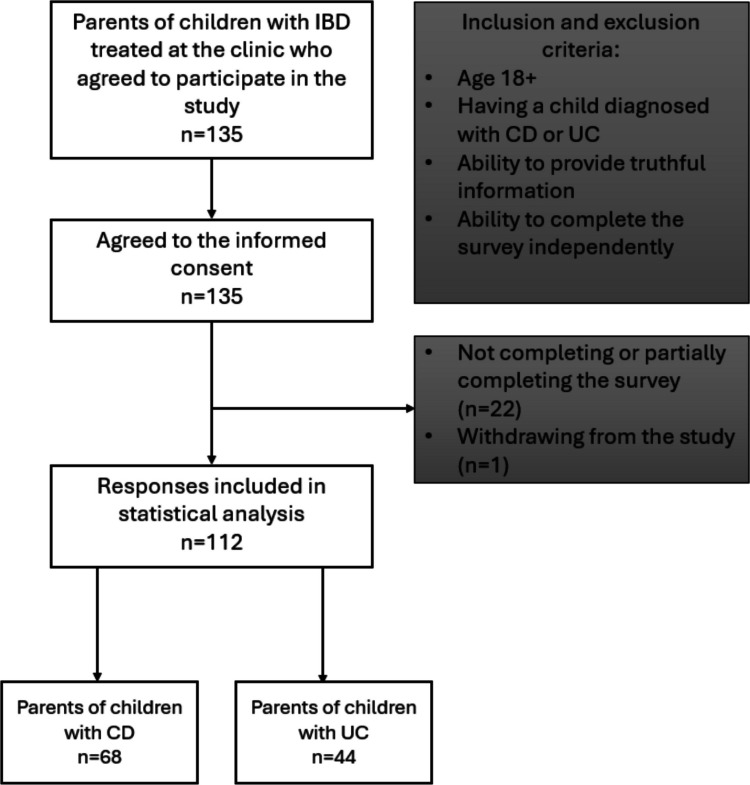
Study design detailing inclusion and exclusion criteria. IBD: inflammatory bowel diseases, CD: Crohn’s disease, and UC: ulcerative colitis

The survey consisted of voluntary completion of an anonymous questionnaire distributed on paper. The questionnaire was provided to parents of patients of the clinic where the study took place. The figure below presents inclusion and exclusion criteria for the study. Subjects were informed of the study's voluntariness and anonymity and that they could withdraw from the study at any time. All subjects gave informed and voluntary consent to participate in the study. After completion, the questionnaires were collected by interviewers, and the results were input into Excel (Microsoft). The questionnaire consisted of demographic and socioeconomic sections. The inclusion and exclusion criteria for the study are shown in Fig. 1. Amounts incurred are presented in Polish Zloty [PLN]; 1 PLN represents a cost of approximately 0.25 euro [EUR, €] and 0.27 dollars [USD, $].

### Demographic section

This section consisted of own questions regarding parents and children age, sex, accommodation, and presence of chronic diseases both in the child and in the family. The children’s diet and the first symptoms of chronic diseases together with the severity of these symptoms at the time of completing the questionnaire, along with the year of diagnosis, were also investigated. The information gathered in the aforementioned questions made it possible to characterize the study population and to exclude results that did not meet the inclusion criteria (Fig. 1). In addition, the evaluation of the first symptoms and duration of the disease made it possible to generally determine the picture of the onset of the disease in the study population.

### Socioeconomic section

Socioeconomic situation was examined by questions concerning taken medications and their monthly costs, private visits to doctors and their costs, the distance travelled to the hospital and the frequency of the need to travel to the hospital, how it was travelled, as well as work activity and absenteeism from work due to the child’s illness.

The data allowed for estimating the monthly costs associated with the child’s illness, both material and non-material. The information collected was used to look for associations between the amount of costs and absenteeism, the type of IBD and other areas of the study.

### Statistical analysis

The collected data were processed using Microsoft Excel and Statistica 13.3 (Wroclaw Medical University’s license, StatSoft Polska Sp. z.o.o., Wroclaw, Poland). In order to avoid estimation error, travel costs were calculated by multiplying the distance given by the parent, the frequency of travel and the cost per kilometre travelled, as defined by Polish law [9]. Mean, standard deviation [SD], and percentages were calculated to represent the data. Normality of distribution was tested using the Shapiro–Wilk test. Because non-parametric data were obtained, relationships between two independent variables were examined with the Mann–Whitney *U* test. To make the results presented transparent, percentages were rounded to decimal places, mean and SD values to hundredths, and *p*-values to thousandths. Values were taken as statistically significant at *p* < 0.05.

## Results

The study included 112 participants, of whom 44 were parents of children with UC and 68 with CD. The annual number of hospitalizations due to Crohn’s disease and ulcerative colitis at the clinic where the study was conducted equals 1425 which stands for 45% of all the hospitalizations taking place. In the last year, 27 new cases of IBD were identified at the clinic, which reflects the incidence of new cases diagnosed in previous years in the Lower Silesia region and country [10]. A notable proportion of patients required treatment with biologic drugs, with 47% of those with CD and 30% of those with UC receiving these drugs. The demographic characteristics of the study sample, shown in Table 1, illustrate the general profile of parents coping with childhood IBD, including age and gender—parents and children—and place of residence.

**Table 1:** Demographic data

	**Ulcerative colitis (UC)** ***n* = 44**	**Crohn’s disease (CD)** ***n* = 68**
Sex of parent, male/female, *n* (%)	16 (36.4)/28 (63.6)	19 (27.9)/49 (72.1)
Sex of the child, male/female, *n* (%)	25 (56.8)/19 (43.2)	43 (63.2)/25 (36.8)
Respondent’s (parent’s) age (at the time of the survey), mean ± SD	44.6 ± 5.8	44.0 ± 5.8
Childs age (at the time of the survey), mean ± SD	14.0 ± 3.7	13.8 ± 3.3
**Residence, *n* (%)**		
Village < 2000 inhabitants	16 (36.4)	17 (25.0)
Small town > 2000, < 100 000 inhabitants	21 (47.8)	36 (52.9)
Big town > 100 000 inhabitants	7 (15.8)	15 (22.1)

Within all families examined in which UC among children were diagnosed only 7 (15.8%) lived in a city with over 100,000 citizens, compared to 16 of residents of villages (36.4%) and 21 from cities with less than 100,000 citizens (47.8%). The average UC patient had to travel 106.3 km while CD patient had to travel 85.2 km to reach the centre where the treatment and medical consultations were performed. In addition, patients with UC travelled this route 17.7 times a year and patients with CD 12.7. The average cost associated with getting to the clinic was calculated and amounted to PLN 215.9 per month for families of patients with UC and PLN 302.3 for parents of patients with CD. It is worth mentioning that in Poland, parents do not bear any direct costs for biological treatment or basic medications used to treat their children, which significantly reduces treatment costs and limits them to indirect costs, such as those examined by us. Medication costs result from the use of supplements and medications other than those recommended by a doctor.

The average duration of the disease was comparable in both groups, accounting for approximately three years. Approximately half of the parents surveyed reported using additional private care related to IBD; these included private medical, dieticians and visits to a psychologist. Among those surveyed, 27.7% of children required visits to a psychologist in private healthcare, which corresponds to more than a half of the declared non-hospital visits. There was no statistically significant difference in the frequency of use of private visits between UC and CD patients (Table 2.), but a significantly statistically significant difference in the cost of private visits was found among them—PLN 312.3 ± 227.2 for UC and PLN 513.3 ± 342.3 for CD with *p*-value 0.004 (Table 3.). Statistically significant differences were also found in the average monthly cost of reaching the treatment centre (Table 3). Defined by polish Labor Code, during a year, the employee can use 2 days paid at 50% for childcare and 5 days unpaid for care of a sick child. Additionally, in accordance with the Act on Social Insurance Cash Benefits in the Event of Sickness and Maternity, a parent is allowed to apply for a childcare allowance in the amount of 80% of the salary for 60 days per year for a child up to 14 years of age and 30 days per year for a child over 14 years of age. Parents whose children suffer from UC spend approximately 32.4 workdays yearly which translates to yearly salary lower 7.07% in the scale of entire year. For parents, whose children has diagnosed CD it is estimated 18 days absence and cut of the salary at the level of approximately 5.78% [11, 12].

**Table 2:** Data related to the treatment of the child’s IBD

	**Ulcerative colitis (UC)** ***n* = 44**	**Crohn’s disease (CD)** ***n* = 68**	*p*-value
Average duration of disease (years), mean ± SD	3.3 ± 2.7	3.2 ± 2.4	-
Number of people using private appointments with specialists, *n* (%)	24 (54.5)	31 (45.6)	0.358
Average distance (km) travelled to treatment centre, mean ± SD	106.3 ± 98.6	85.2 ± 57.3	0.265
Average annual number of visits to a treatment centre, mean ± SD	17.7 ± 13.1	12.7 ± 8.1	0.057
Average number of days per month of parent absence related to child’s IBD, mean ± SD	2.7 ± 3.4	1.5 ± 1.4	0.019*

Values in bold indicate statistically significant differences at *p*<0.05

**Table 3:** Average monthly costs in PLN related to a child’s IBD

	Ulcerative colitis *n* = 24	Crohn’s disease *n* = 31	*p*-value
Average monthly cost of visits to specialists among those using private visits, PLN, mean ± SD	312.3 ± 227.2	513.3 ± 342.3	0.004*
	Ulcerative colitis *n* = 44	Crohn’s disease *n* = 68	*p*-value
Monthly cost to parents of medication related to their child's IBD, PLN, mean ± SD	254.5 ± 209.9	304.9 ± 266.2	0.314
Average monthly cost incurred to reach the clinic, PLN, mean ± SD	215.9 ± 224	302.3 ± 276.2	0.023*

*PLN* Polish zloty, *1 PLN* 0.25 EUR

Values in bold indicate statistically significant differences at *p*<0.05

The issue of selecting an appropriate diet for a patient with IBD is an important aspect of treatment. In the group examined in the clinical trial with UC (*n* = 44), the use of a diet eliminating lactose was declared by 14 parents (31.8%), eliminating gluten by 7 (15.9%), including 3 (6.8%) parents who declared the elimination of both gluten and lactose. Among people with CD (*n* = 68), the use of a diet eliminating lactose was declared by 21 parents (30.9%), eliminating gluten by 7 (10.3%), including 4 (5.9%) parents who declared the elimination of both gluten and lactose. In total, in the study group, 65 patients declared their diet as easily digestible. The information on dietary practices was obtained from an open-ended question, allowing respondents to freely describe the type of diet used.

Among patients with ulcerative colitis (*n* = 44), apart from biological treatment, mesalazine (24 patients, 58.5%), azathioprine (14 patients, 34.1%) and methylprednisolone (5 patients, 12.2%) were most commonly used in various combinations. In the group of patients with Crohn’s disease (*n* = 69), the most commonly used drugs were mesalazine (41 patients, 59.4%), azathioprine (27 patients, 39.1%) and sulfasalazine (14 patients, 20.3%), in various therapeutic configurations. In the group of patients with Crohn’s disease not treated with biological agents (*n* = 35), treatment included azathioprine (23 patients, 65.7%), mesalazine (19 patients, 54.3%) and sulfasalazine (10 patients, 28.6%), used in various combinations. The average annual number of visits to the centre among patients receiving biological treatment was 14.06 ± 7.7, while among patients without biological treatment it was 14.39 ± 11.0. Patients using private consultations had an average of 15.53 ± 11.03 visits per year, while those not using private consultations had 13.88 ± 10.71 visits per year.

## Discussion

In this survey study, we analysed the demographic data of paediatric patients with IBD, and the costs incurred by the patient’s family for the treatment of the child and other factors influencing the generation of costs related to treatment or requiring a large amount of time spent on the care of a paediatric patient with UC or CD. For almost 11 months, surveys were collected from parents of patients at a paediatric gastroenterology clinic in Wroclaw. We analysed trends in the results related to the costs related to IBD and both medical and paramedical decisions that may affect the patient’s condition.

### Transportation cost and demographic data

The data mentioned above indicate that significant expenses are incurred each month to transport the child. This may suggest a small number of centres specializing in providing professional support to patients with IBD, whereas the problem is widespread in both low and highly urbanized areas. This may also potentially affect the reduced level of diagnosis of IBD in paediatric patients compared to the actual state or reduced level of patient care, as is also the case in other disease entities [13, 14]. Numerous reports have identified transportation costs as a barrier in treatment [15, 16]. The issue of transport-related fees borne by parents of children with IBD is insufficiently studied and difficult to determine [17]. Sums present in the literature are, for example, €1 per month in India or €7.4 in China [18, 19]. According to a study in the USA, most parents incurred costs in the range of $33 to $166 per month [20]. In the European Union, out-of-pocket costs are considered difficult to estimate, but the authors point out that they represent a significant expense and that there is considerable variation between countries. The overall costs incurred for treatment in the EU exceed those determined by our study [21, 22]. Despite the fact that, according to our study, parents of children with CD tend to travel to treatment centres with similar frequency and over similar distances as parents of UC patients, the increased transportation costs among the former group may be related to more frequent visits to private specialists. Studies conducted in the past confirm the higher costs incurred by parents of children with CD and indicate that transportation costs contribute significantly to the overall costs incurred for treatment [23, 24].

### Dietary choices among bowel disease patients

The choice of diet has a significant impact on the functioning of the digestive system [25]. Depending on the selected diet, the amount of secreted enzymes, hormones, mucus, and the composition of the intestinal microbiota may change [26, 27]. These factors play a significant role in the metabolic functioning of the digestive system, and consequently, these changes can influence the activity of IBD [27]. Studies show that a highly processed diet rich in carbohydrates and fats and low in fibre correlates with exacerbations of IBD. This is due to the increased production of proinflammatory cytokines such as IL-6, IL-1β, IL-10, TNF-α, and C-reactive protein (CRP) [26, 28]. Depending on the type of food consumed, the Dietary Inflammatory Index (DII) can be used to estimate the potential impact of dietary patterns on the clinical condition of patients with IBD [28]. When remission is achieved, the main goal of treatment is to maintain it for as long as possible. This can be supported in CD by dietary interventions such as exclusion diets, while in UC such an effect is less pronounced. Nevertheless, maintaining an adequate diet in these patients remains important [29].

Considering the above, nutrition in CD and UC may have a significant impact on disease course and patient outcomes. Among the nutritional strategies applied in the management of paediatric inflammatory bowel disease (IBD), the Crohn’s Disease Exclusion Diet (CDED) represents the most significant and evidence-based dietary intervention. CDED is a structured dietary program developed to induce remission in Crohn’s disease by excluding specific food components and emphasizing defined whole-food items. It is typically implemented in clearly defined phases, with the first phase being the most restrictive and gradually liberalised over time [30–32]. Because its therapeutic effect depends on a phased approach rather than a simple exclusion of specific components, CDED may have been reported less frequently by parents, as it is not perceived as a straightforward elimination diet (e.g., gluten-free). Furthermore, since dietary information in this study was collected through open-ended questions, parents may not have explicitly indicated the use of CDED. In our study (see Table 2), the mean time since diagnosis was approximately three years. It is possible that families who had previously implemented this dietary regimen became accustomed to its modified form over time and no longer regarded it as a distinct therapeutic diet, but rather as the child’s habitual dietary pattern.

Another principal factor that may influence dietary choices is the cost associated with implementing such regimens, as CDED often requires the use of more specialized food products and nutritional supplements. In Poland, a package of Modulen costs €15.3 without reimbursement and €0.74 with reimbursement. The only indication for reimbursement of the product is the induction of remission in children and adolescents over the age of five with active Crohn’s disease, meaning that patients who do not meet these criteria must pay the full price. Depending on the child’s age and caloric requirements, the monthly cost of full supplementation may reach up to €459, assuming a daily caloric intake of 2000 kcal. A study conducted in Spain indicated that, under their economic conditions, the cost of using this diet does not exceed typical dietary expenses [33]; however, in the context of the Polish economic and legal environment, these costs may substantially exceed the average food budget for a healthy child.

### Private medical appointments

Among children with UC, 24 (54.5%) required the use of private visits to specialists, while in CD, the number was 31 (45.6%). A common reason for using private visits to a specialist in Poland is the shorter waiting time for an appointment compared to the public health care system [34]. This situation is due to one of the main problems of the health care system in Poland—the low number of doctors per one thousand inhabitants [35]. In addition, the number of specialists—paediatric gastroenterologists—is low—in Poland, as of 2024, the number was 137, while the number of paediatric IBD patients was 3794 [36]. The use of private visits increases family expenses. Due to the relapsing–remitting nature of these diseases, visits to the gastroenterologist are essential for their monitoring. Furthermore, the increasing incidence in the paediatric population will undoubtedly lead to an increase in the total costs incurred by families with a child with IBD [16, 37]. These costs are further increased when the disease is unstable [20].

### Inflammatory bowel disease and other chronic paediatric diseases

The cost of treating children with IBD is multifaceted compared to other diseases. According to a Canadian study, the average cost of treatment one year after diagnosis is $33,533, whereas, for example, the average annual cost of treating epilepsy in the US is $1853, which is repeatedly lower [38, 39]. Parents of children with UC and CD similarly show higher absenteeism from work, as do, for example, parents of asthmatics [40]. Also, the need for a more restrictive diet forces parents to make a greater time commitment [41]. At the same time, this leads to a reduction in parents’ leisure time or time spent travelling [42]. It follows that the child’s therapy should be combined with simultaneous psychological support for the parents.

### Time input in disease treatment

IBD are chronic diseases that require a large investment of time for both the patient and the patient’s family to treat them [43]. This is another factor that not only imposes additional costs on the treatment of patients but also increases the amount of time spent by the patients’ parents on visits related to treatment. On average, parents miss 2.7 days from work per month for a patient with UC and 1.5 days for a child with CD. This may also contribute to a decrease in the household economy due to missed workdays. However, the illnesses of paediatric patients do not have the same impact on the time spent by adults on health care; depending on the child’s condition and the severity of their illness, this time may be negligible or exceptionally long [44–46]. IBD, in addition to limiting the time patients’ parents spend at work, significantly reduce the time affected children spend in educational institutions and with their peers [47]. This may be a significant factor contributing to the reduction in the quality of life among patients with CD and UC, which has been proven in studies [48]. This trend not only persists in young patients’ parents, but also patients with IBD themselves significantly more often miss workdays than healthy population and statistically more often remain unemployed [49].

### Biological therapy

A major breakthrough in the treatment of IBD in children has been the introduction of biologic drugs. They provide a longer remission compared to conventional drugs and allow lesions to heal and the disease to be better controlled [50]. The main drugs used in biological therapy in children are anti-TNF antibodies (infliximab, adalimumab). Anti-integrins, anti-IL-12 and anti-IL-23 antibodies, among others, are also used [51]. Of those surveyed, 47% of CD patients and 30% of UC patients were treated with biologics, representing a considerable proportion of clinic visits. In recent years, there has been an increase in the percentage of biologic therapy in the paediatric IBD population [52]. Biological therapy has become the European standard, but new therapeutic approaches for the treatment of CD and UC are being sought all the time. Biosimilars, which are akin to a biologic drug but differ in the manufacturing process and post-translational processing, are being investigated. Research shows that their safety is maintained, with a possible reduction in the cost of therapy [53]. Many other therapies are being studied with adults, and in the longer term, there is the possibility of including children in studies [54]. On the other hand, biological therapies have several disadvantages. The high expenses stem from both the drugs themselves and the associated healthcare costs, such as hospitalizations for monitoring and managing potential adverse effects like anaphylaxis, which are often omitted in estimating the cost of biological therapy. For example, infliximab and other anti-TNF therapies are among the costliest outpatient medications, accounting for a significant portion of IBD treatment expenses [16]. Beyond financial concerns, biologic therapies can lead to side effects, including immunogenic reactions, infections, and rare but severe complications. The need for close monitoring increases both the complexity and the cost of care [55]. Exploring how to optimize biologic use, including patient stratification and dose adjustments, may help mitigate the financial and clinical burdens associated with these treatments. The hope to reduce costs and non-financial outlays is to administer the drugs subcutaneously, which offers the possibility of outpatient treatment and only periodic follow-ups at the clinic without the need for hospitalization each time the drug is administered. This solution was introduced mainly due to complaints raised by parents and is a viable response to the problems they face with their children's illness.

### Limitations

Several important limitations of this study should be noted. The number of respondents was limited, so a greater sample size should be considered in further research. Another important limiting factor is the declarative nature of the study, which implies that only estimated absenteeism and expenses incurred by parents could be obtained. Socioeconomic status could be examined by assessing several indicators: the ownership or rental of the living area, possession of a car, usage of public transport, the education system chosen for children and parents’ educational degree. In a future study, it would be beneficial to broaden the condition of the patient and deepen the medical history. Considering the above, we suggest using highly specific questions about material and non-material costs incurred in order to obtain the most reliable results possible. Future studies should also consider adding a group of patients with other chronic diseases to compare costs between conditions. It is recommended in future studies to determine how much the parents of patients think they incur in costs related to their children’s illness. Due to the limitations mentioned above, the study conclusions cannot be considered definitive.

## Conclusions

Taking care of children suffering from IBD poses many difficulties and costs to the parent. Both CD and UC patients require significant medical expenses, as well as many days of care in the hospital and in private facilities. Both transportation and the cost of medications and visits to specialists represent a significant expense for caregivers. In addition, caring for the patient generates significant absenteeism from work.

Based on the findings of the study, efforts should be made to increase financial and psychological support for caregivers of IBD patients. There is also a need to implement systemic solutions to reduce the costs incurred. A solution to the problem could be an increase in healthcare funding for patients with IBD. We recommend that parents of patients with UC and CD should be given particular support regarding the financing of their children’s treatment and the continuation of their occupational activities. In future studies, it would be a valuable addition to examine additional factors influencing costs incurred by caregivers. Those factors should be examined using more precise methods.

## Data Availability

Data are contained within the article.
